# An efficient two-step subcellular fractionation method for the enrichment of insulin granules from INS-1 cells

**DOI:** 10.1007/s41048-015-0008-x

**Published:** 2015-08-07

**Authors:** Yan Chen, Zhiping Xia, Lifen Wang, Yong Yu, Pingsheng Liu, Eli Song, Tao Xu

**Affiliations:** National Laboratory of Biomacromolecules, Institute of Biophysics, Chinese Academy of Sciences, Beijing, 100101 China; University of Chinese Academy of Sciences, Beijing, 100049 China

**Keywords:** Insulin, Immature insulin secretary granules (iISGs), Mature insulin secretary granules (mISGs), Subcellular fractionation, Density gradient

## Abstract

Insulin is one of the key regulators for blood glucose homeostasis. More than 99% of insulin is secreted from the pancreatic β-cells. Within each β-cell, insulin is packaged and processed in insulin secretary granules (ISGs) before its exocytosis. Insulin secretion is a complicated but well-organized dynamic process that includes the budding of immature ISGs (iISGs) from the *trans*-Golgi network, iISG maturation, and mature ISG (mISG) fusion with plasma membrane. However, the molecular mechanisms involved in this process are largely unknown. It is therefore crucial to separate and enrich iISGs and mISGs before determining their distinct characteristics and protein contents. Here, we developed an efficient two-step subcellular fractionation method for the enrichment of iISGs and mISGs from INS-1 cells: OptiPrep gradient purification followed by Percoll solution purification. We demonstrated that by using this method, iISGs and mISGs can be successfully distinguished and enriched. This method can be easily adapted to investigate SGs in other cells or tissues, thereby providing a useful tool for elucidating the mechanisms of granule maturation and secretion.

## Introduction

Insulin is one of the key regulators for blood glucose homeostasis. Abnormal insulin secretion is considered an essential factor in the progression of diabetes (Weir et al. 2001). More than 99% of insulin is secreted from the pancreatic β-cells (Rhodes 2000). Within each β-cell, insulin is stored in specified organelles known as insulin secretary granules (ISGs), and is released via a regulated secretory pathway. Insulin biogenesis initiates with the synthesis of preproinsulin in the rough endoplasmic reticulum (ER) and the conversion of preproinsulin to proinsulin. Proinsulin begins to be packaged in the trans-Golgi network (TGN) and is sorted into immature ISGs (iISGs). These iISGs gradually become acidic, and proinsulin undergoes proteolytic cleavage, resulting in the formation of mature bioactive insulin and C-peptide (Docherty and Hutton 1983; Orci et al. 1986; Smeekens et al. 1992; Colomer et al. 1996; Davidson 2004; Steiner et al. 2009). During this maturation process, insulin is crystallized in the form of dense core, and unwanted cargoes and membrane proteins are removed and transported either back to the TGN or endo/lysosome systems (Kim et al. 2006). The newly formed mature ISGs (mISGs) are transported to the plasma membrane to be ready for release in response to calcium stimulation (Borgonovo et al. 2006).

Although SGs have been known for almost 70 years, many questions, including how SG-specific components are segregated from molecules destined to different organelles and how SGs mature, remain elusive. Therefore, it is necessary to separate iISGs and mISGs to identify their characteristics and protein contents. An efficient isolation of these two organelles is the first and most important step for the subsequent characterizations. However, because ISG maturation is a highly dynamic process, it is extremely difficult to separate iISGs and mISGs. Several previous studies reported methods to isolate ISGs, but the samples isolated in these works were total ISGs, which contained both iISGs and mISGs (Konrad et al. 1995; Iezzi et al. 1999; Brunner et al. 2007). Another group recently reported the development of a three-step gradient purification procedure combined with Stable Isotope Labeling with Amino acids in Cell culture (SILAC) to further characterize the ISG proteins. However, the fraction purities of iISGs and mISGs were not well verified (Schvartz et al. 2012).

In this study, we developed a modified two-step subcellular fractionation method to simultaneously enrich high-purity iISGs and mISGs from cultured INS-1 cells, a rat-derived β-cell line. We used a primary discontinuous OptiPrep gradient to isolate two fractions (Fractions 6 and 8) in which insulin was abundant. Immunoblotting results and fraction densities indicated that Fractions 6 and 8 were iISGs and mISGs, respectively. Further enrichment of iISGs and mISGs was accomplished using Percoll solution.

## Results

### The two-step enrichment protocol for isolating iISGs and mISGs

Accurate analyses of ISGs are always based on the quality of iISG and mISG enrichment. For the efficient isolation of iISGs and mISGs, we employed an OptiPrep density gradient to enrich iISGs and mISGs simultaneously. The efficient two-step method was established. Briefly, (1) the cultured cells were homogenized in Buffer A by nitrogen cavitation to obtain whole cells (WC), and after centrifugation, the postnuclear supernatant (PNS) was obtained; (2) the PNS was loaded on top of an initial discontinuous OptiPrep gradient with five concentrations; (3) the fractions were collected and examined by ELISA and Western blot after ultra-centrifugation; (4) the insulin containing fractions were added in certain concentration Percoll buffer followed by ultra-centrifugation; and (5) the fractions containing lower levels of contaminants were collected as iISGs or mISGs.

### OptiPrep fractionation of subcellular components

**Fig. 1:**
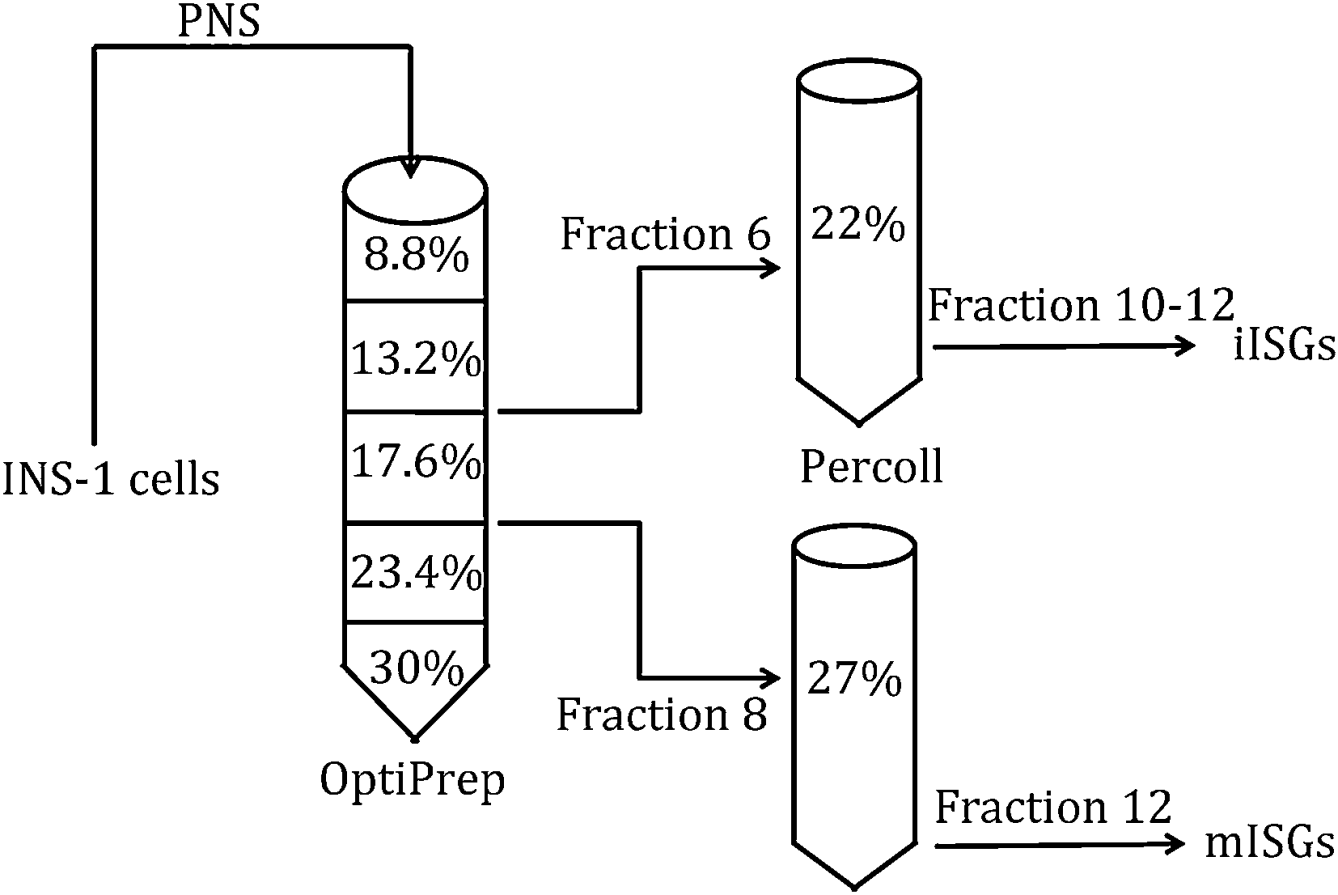
Schematic illustrating the isolation procedures of iISGs and mISGs from INS-1 cells. Briefly, the INS-1 cell postnuclear supernatant was loaded on top of an OptiPrep gradient consisting of five layers of OptiPrep at varying concentrations. After ultra-centrifugation, the two interfaces between 13.2% and 17.6% and 17.6% and 23.2% were collected as Fractions 6 and 8. Subsequently, these two fractions were further fractionated using 22% and 27% Percoll, respectively. Fractions 10–12 and Fraction 12 of 22%/27% Percoll were collected as the final iISG and mISG fractions

**Fig. 2:**
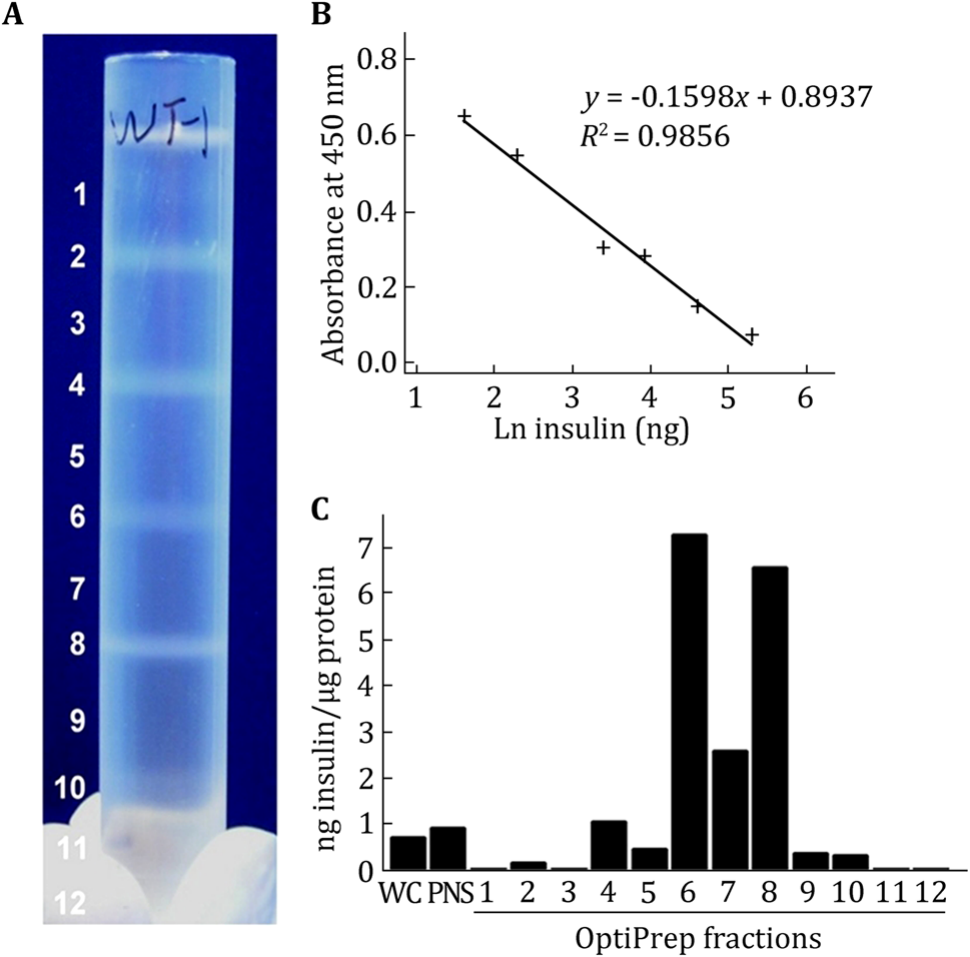
Analyses of the fractions from the first step of subcellular fractionation. **A** An example of the fraction distribution after centrifugation. **B** Standard curve for competitive insulin ELISA. **C** Insulin quantification in each of the 12 fractions obtained from the OptiPrep gradient

To isolate iISGs and mISGs, the first step, the enrichment of subcellular fractions, was performed. PNS collected from cultured INS-1 cells was separated on a discontinuous OptiPrep density gradient including five concentrations (8.8%, 13.2%, 17.6%, 23.4%, and 30%) (Fig. 1). Twelve fractions were individually collected from the top of the OptiPrep gradient (Fig. 2A). To determine which fractions were the enriched iISGs or mISGs, the insulin content was quantified using an ELISA assay. Insulin concentrations in this experiment were in the range of 5–200 ng of the standard curve for a competitive ELISA (Fig. 2B). Fractions 6 and 8 were observed to exhibit the highest levels of insulin (Fig. 2C). It should be mentioned here that the insulin antibody used in the ELISA assay (Sigma, I2018) has high cross-reactivity with proinsulin. Therefore, both iISGs, which contained a large proportion of proinsulin, and mISGs, which contained a large proportion of insulin, showed high insulin levels. Previous studies had reported that iISGs differ from mISGs in size and density, with mISGs acquiring higher density and larger size after maturation (Tooze et al. 1991; Tooze and Stinchcombe, 1992). In the OptiPrep density gradient, the interface of Fraction 6 was between 13.2% and 17.6%, and the interface of Fraction 8 was between 17.6% and 23.4%, indicating that Fraction 8 was denser than Fraction 6. Therefore, iISGs were considered to be enriched in Fraction 6 and mISGs in Fraction 8.

### Validation of iISG and mISG fractions

**Fig. 3:**
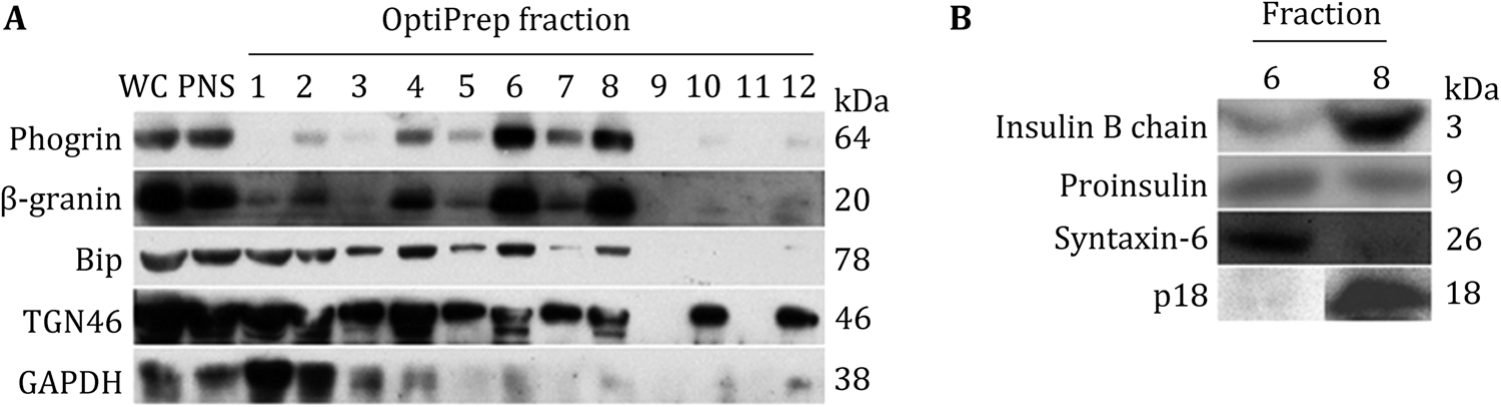
Western blot assessment of the 12 fractions from OptiPrep gradient. **A** Western blot analyses of whole cell lysate (WC), postnuclear supernatant (PNS), and the 12 fractions using antibodies against phogrin, β-granin, Bip, TGN46, and GAPDH. **B** Western blot analyses of Fraction 6 and Fraction 8 using antibodies against insulin, proinsulin, Syntaxin-6, and p18. Equal amounts of proteins from each fraction were loaded onto SDS-PAGE gels

To characterize each of the 12 fractions isolated from the first step, equal amounts of proteins from each fraction were separated by SDS-PAGE followed by Western blot using antibodies against various markers of cellular compartments, including phogrin, which is a classical ISG-associated membrane protein (Wasmeier and Hutton, 1996); β-granin, which is a peptide that is co-secreted with insulin and is considered to be a marker for ISG (Hutton et al. 1985); Bip, which is a HSP70 molecular chaperone located in the ER lumen and is hence considered a marker for the ER (Hendershot et al. 1994); TGN46, which is a marker for the TGN (McCrossan et al. 2001); and GAPDH, which is a marker for the cytoplasm. As shown in Fig. 3A, phogrin and β-granin were most strongly expressed in Fractions 6 and 8, consistent with the ELISA results, while GAPDH was not detected in Fractions 6 and 8, indicating that these two fractions were not contaminated by cytosolic proteins. However, Bip and TGN46 were also detectable in Fractions 6 and 8, indicating these two fractions were contaminated by ER and Golgi to a small extent.

To further determine iISGs and mISGs, we detected insulin, proinsulin, p18, and Syntaxin-6 in Fractions 6 and 8. p18 is a product of SgII that is processed at several sites by PC2 and accumulates in mISGs; it can be detected using an anti-p18 antibody recognizing only p18 but not larger SgII precursors (Dittie and Tooze 1995; Ahras et al. 2006). Syntaxin-6 functions in the granule maturation process and is localized to TGN and iISG membranes, whereas mISGs have lower levels of this protein (Bock et al. 1996; Klumperman et al. 1998; Wendler et al. 2001). As shown in Fig. 3B, the levels of insulin and p18 in mISGs were higher in Fraction 8 than those in Fraction 6, while less proinsulin and Syntaxin-6 were detected in Fraction 8 than in Fraction 6. These results suggest that iISGs are enriched in Fraction 6 and mISGs in Fraction 8.

### Further enrichment of iISGs and mISGs

**Fig. 4:**
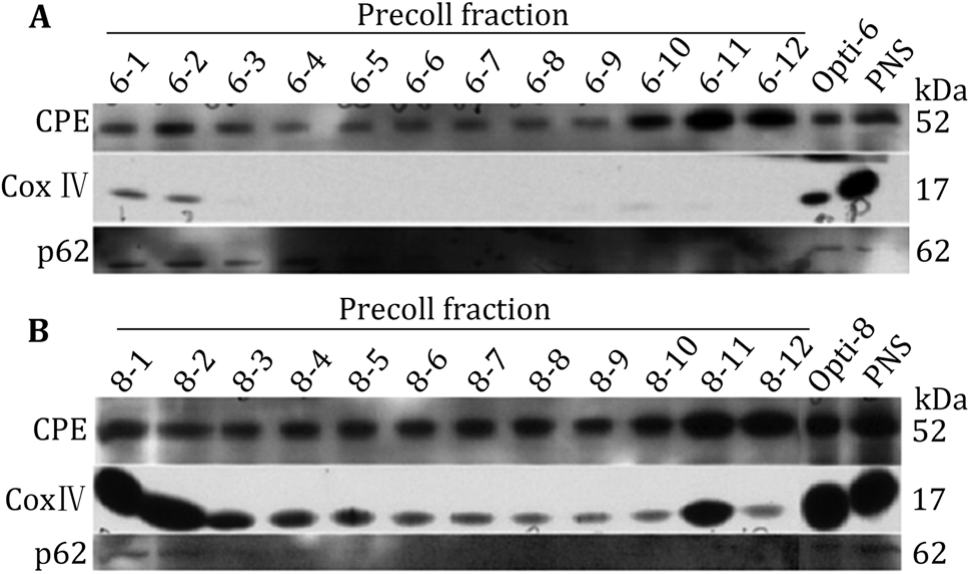
Western blot analyses of Percoll fractions 1–12 of OptiPrep Fraction 6 (Opti-6) and OptiPrep Fraction 8 (Opti-8) with antibodies directed against CPE, CoxIV, and p62; Opti-6/8 and PNS were used as controls. **A** Percoll fractions 1–12 of OptiPrep Fraction 6 (opti-6); **B** Percoll fractions 1–12 of OptiPrep Fraction 8 (Opti-8). Equal volumes of Percoll fractions were loaded onto SDS-PAGE gels

Although iISGs and mISGs were successfully enriched in Fractions 6 and 8, each of the fractions had some contaminations. To further enrich iISGs and mISGs, OptiPrep Fractions 6 and 8 were subsequently separated using Percoll solutions. Several concentrations of Percoll, including 20%, 22%, 25%, 27%, and 30%, were tried first (data not shown), and finally, we determined that the 22% Percoll solution was suitable for Fraction 6 and the 27% solution for Fraction 8. Twelve Percoll fractions from Fraction 6 or Fraction 8 were collected. We performed Western blotting to validate each of these fractions using antibodies against three markers including CPE, which is a prohormone-processing enzyme localized to peripheral membrane of ISGs and is used as a marker for ISG; CoxIV, which is a marker for mitochondria (Nakagawa and Miranda 1987); and p62, which is a marker for ER (Mundy and Warren, 1992). As shown in Fig. 4, CPE was successfully enriched, especially in P10, P11, and P12. CoxIV was absent from P6-3 to P6-12, whereas it was observed in all fractions of P8, indicating that this fraction was contaminated by mitochondria. p62 was barely detectable in both Fractions 6 and 8. Western blot analysis showed that in P6-10 to P6-12, CoxIV and p62 were absent, whereas CPE was present at high levels (Fig. 4A), suggesting that these two fractions were the iISGs fractions. mISGs were well enriched in P8-12 with low levels contaminants. Therefore, we could enrich iISGs in fractions P6-10 to P6-12 and mISGs in fraction P8-12.

## Discussion

Subcellular fractionation has been widely used for the analyses of various intracellular organelles and complexes. In this study, we developed an efficient two-step fractionation strategy for the enrichment of iISGs and mISGs, in which an initial discontinuous OptiPrep gradient was applied, followed by a Percoll solution. This method generated highly enriched fractions of iISGs and mISGs as demonstrated by Western blotting against different intracellular organelle markers.

Our major challenge was the simultaneous isolation of iISGs and mISGs, which are two separate organelles that share many properties. Using a discontinuous OptiPrep gradient, we successfully separated total ISGs into two fractions, though ER and TGN contaminants were detected in both of them. After the second step, Percoll solution enrichment, we obtained purer iISGs and mISGs fractions, but mitochondria contaminants still existed in the mISG fraction. Generally, complete purification of an organelle by subcellular fractionation is impossible (Huber et al. 2003). There are two main possible reasons for this. First, intracellular organelles are generally highly dynamic entities; this is precisely the case for iISGs and mISGs, the properties and protein contents of which continuously change during the granule maturation process, from budding at the TGN to release at the plasma membrane (D’Amico et al. 2001). This may explain why our enriched fractions were contaminated by TGN contents. Second, a few subcellular structures share similar physical properties in density gradients, which make it difficult to completely separate them. In our experiment, for example, ER contamination was detected in the first step fractionation due to the broad density range of ER-derived membranes (Mast et al. 2010); mitochondria, which are similar to mISGs in density, were still observed in the secondary mISG fraction.

Although the subcellular organelle purification method based on density gradients makes it difficult to avoid contamination by unwanted components, it is still a common and efficient method to enrich our interested organelles. Previously, two proteomic studies were conducted to enrich ISGs followed by proteomic analysis. In the first work, they only enriched total ISGs, without further distinguishing between iISGs and mISGs (Brunner et al. 2007). In the second study, they used a three-step gradient purification procedure to obtain both iISGs and mISGs; however, the properties and purity of the final fractions were not well characterized (Schvartz et al. 2012). Here, we developed a simpler enrichment method and achieved better separation, as verified by many specific markers. Specifically, we used an insulin ELISA and Western blotting against insulin, proinsulin, Syntaxin-6, and p18 to determine the iISG and mISG fractions, and we used Western blot against phogrin, β-granin, Bip, TGN46, GAPDH, CPE, CoxIV, and p62 to determine the purity of our enriched fractions.

ISGs are pivotal organelles of pancreatic β-cells and represent key participants of glucose homeostasis. It is important to acquire highly purified iISG and mISG fractions for further proteome analyses and biochemical studies to increase our knowledge of ISG biogenesis, maturation, and exocytosis. More complete elucidation of these processes is indispensable for the understanding of β-cell function and abnormalities in pathologies, such as type 2 diabetes.

In conclusion, we described a reliable, reproducible, and easy method for the enrichment of mISGs and iISGs from INS-1 cells. This method can be easily adapted to investigate SGs in other cells or tissues.

## Materials and methods

All chemicals were purchased from Sigma-Aldrich with the highest purity available, unless otherwise stated. Percoll solution was obtained from GE Healthcare (17089109). The OptiPrep solution was obtained from Sigma (D1556). A chicken antibody against phogrin was a gift from Dr. Joseph K. Angleson (University of Denver, Denver, USA). The mouse antibody against CPE was from BD (1:2000, 610758). The rabbit antibody against TGN46 was from Sigma (1:1000, T7576). The mouse monoclonal antibody against p18 was from Abcam (1:1000, ab20245). The mouse antibody against β-granin was from Abcam (1:1000, ab81606). The mouse antibody against p62 was from Transduction Laboratories (1:1000, P20120). The mouse antibody against GAPDH was from Chemicon (1:1000, MAB374). The mouse antibody against Bip was from BD (1:1000, 610979). The mouse antibody against Syntaxin-6 was from Abnova (1:1000, H00010228-M01). The guinea pig antibody against insulin was from Abcam (1:1000, ab7842). The mouse antibody against proinsulin was from HyTest (1:1000, 2PR8). The antibody against CoxIV was a gift from Dr. Pingsheng Liu (Institute of Biophysics, Beijing, China).

### Cell culture

INS-1 cells were cultured in regular RPMI-1640 medium (Gibco) supplemented with 11.1 mmol/L D-glucose, 10% fetal bovine serum (Gibco), 1 mmol/L sodium pyruvate (Sigma), and 50 μmol/L mercaptoethanol (Sigma) and maintained at 37°C and 5% CO_2_ in a humidified incubator.

### Isolation of insulin secretory granules

iISGs and mISGs were obtained by subcellular fractionation from INS-1 cells. All the following procedures were performed at 4 °C. Briefly, two 150-mm plates of INS-1 cells were washed once with ice-cold PBS and scraped in ice-cold PBS with 1 mmol/L PMSF. Cells were then homogenized in 5 mL of Buffer A (0.3 mol/L sucrose, 1 mmol/L EDTA, 1 mmol/L MgSO_4_, 10 mmol/L MES-KOH, pH 6.5) containing 1 mmol/L PMSF by nitrogen cavitation for 15 min at 450 psi on ice. The homogenate (whole cell) was centrifuged at 1000 *g* for 5 min to remove unbroken cells and nuclear debris. The supernatant was collected and loaded on top of a discontinuous Optiprep gradient composed of 5 layers (2 mL 30%, 2 mL 23.4%, 2 mL 17.6%, 2 mL 13.2%, and 2 mL 8.8%). 30% OptiPrep was prepared as stock in Buffer B (2 mmol/L EGTA, 20 mmol/L MES-KOH, pH 6.5), and the rest were made by diluting 30% OptiPrep with Buffer A containing 1 mmol/L PMSF in a SW40 tube, and the sample was centrifuged at 100,000 *g* for 75 min. On the basis of visible interface, each fraction was collected in appropriate 600 μL. Fraction 6 (interface between 13.2% and 17.6%) and Fraction 8 (interface between 17.6% and 23.4%), which contained the most insulin, were considered to be the iISG and mISG fractions, respectively. In the second gradient centrifugation, we used Percoll instead of Optiprep (Sigma, D1556). 2 mL diluted Fraction 6 was mixed with 10 mL 22% Percoll solution (2.2 mL Percoll diluted to 10 mL with modified Buffer A containing 0.27 mol/L sucrose instead of 0.3 mol/L) and 2 mL diluted Fraction 8 was loaded on top of 10 mL 27% Percoll solution (2.7 mL Percoll diluted to 10 mL with Buffer A). Then the Percoll samples were centrifuged at 35,000 *g* for 45 min. The top 1 mL was discarded, and the remainder was split in ~0.9 mL aliquots for the following experiments.

### Quantification of insulin levels in isolated insulin secretory granules

The insulin contents of the ISG fractions were measured using a competitive ELISA assay. Before measurement, 0.05 g/mL insulin (Sigma, I0516) was used to coat a Nunc immuno-module 96-well plate overnight at 100 μL/well. Stepwise insulin concentrations from 0 to 400 ng were mixed and preincubated with 30 ng of mouse anti-insulin antibody (Sigma, I2018) in 100 μL of PBST (PBS with 0.05% tween-20) in a 1.5-mL tube for 1 h. Meanwhile, the coated plate was blocked by 0.2% BSA in PBST for 1 h after washing 3 times with 300 μL/well in PBST. The mixture of insulin and the anti-insulin antibody was added in triplicate to the insulin-coated wells, and the plate was incubated for 1 h. After incubation, the plate was washed three times with PBST, 300 μL/well. The plate was then incubated with Biotin-SP-conjugated goat anti-mouse IgG (H + L) (Jackson ImmunoResearch, code number: 115-065-003, 1:30,000) for 1 h, followed by another incubation with peroxidase-conjugated streptavidin (based on the recommended concentration) for 1 h after washing six times with 300 μL/well of PBST. Finally, 50 μL of peroxidase substrate (TMB) was added, and the plate incubated for 10 min followed by addition of 50 μL of 0.3 mol/L HCl to stop the reaction. Absorbance was measured at 450 nm.

### Western blot

Protein concentration was determined with the bicinchoninic acid assay (Thermo Scientific, 23227). Proteins were separated by SDS-PAGE (insulin and proinsulin were separated in Novex 4–12% Bis–Tris gel (Life Technologies, 12050371)) and were then transferred to PVDF membranes. The membranes were first blocked for 1 h with 5% non-fat dry milk in TBST, incubated for 1–2 h with primary antibody, washed 4 times for 5 min each time in TBST, incubated for 1 h with the appropriate peroxidase-conjugated secondary antibody, washed 4 times for 7 min each time in TBST, and then developed photographically by ECL.
